# Supplementary material for: Resolving the full spectrum of human genome variation using Linked-Reads
Source: Genome Res. 2019 Apr;29(4):635–45. doi: 10.1101/gr.234443.118 (PMC6442396; doi:10.1101/gr.234443.118)
Supplement: Supplemental Material [file supp_gr.234443.118_Supplemental_longranger-2.2.zip › longranger-2.2.tar.bz2!/lr2.2-public/lib/stan/cmdstan/stan/doc/api/html/cumulative__sum_8hpp_source.html]

Stan: src/stan/math/matrix/cumulative\_sum.hpp Source File


|  |  |
| --- | --- |
|  | Stan  2.5.0  probability, sampling & optimization |


- Main Page
- Related Pages
- Namespaces
- Classes
- Files

- File List
- File Members

All Classes Namespaces Files Functions Variables Typedefs Enumerations Enumerator Friends Macros Pages

- src
- stan
- math
- matrix

cumulative\_sum.hpp

Go to the documentation of this file.

1 #ifndef STAN\_\_MATH\_\_MATRIX\_\_CUMULATIVE\_SUM\_HPP

2 #define STAN\_\_MATH\_\_MATRIX\_\_CUMULATIVE\_SUM\_HPP

3

4 #include <vector>

5 #include <stan/math/matrix/Eigen.hpp>

6

7 namespace stan {

8  namespace math {

9

21  template <typename T>

22  inline std::vector<T>

23  cumulative\_sum(const std::vector<T>& x) {

24  std::vector<T> result(x.size());

25  if (x.size() == 0)

26  return result;

27  result[0] = x[0];

28  for (size\_t i = 1; i < result.size(); ++i)

29  result[i] = x[i] + result[i-1];

30  return result;

31  }

32

47  template <typename T, int R, int C>

48  inline Eigen::Matrix<T,R,C>

49  cumulative\_sum(const Eigen::Matrix<T,R,C>& m) {

50  Eigen::Matrix<T,R,C> result(m.rows(),m.cols());

51  if (m.size() == 0)

52  return result;

53  result(0) = m(0);

54  for (int i = 1; i < result.size(); ++i)

55  result(i) = m(i) + result(i-1);

56  return result;

57  }

58  }

59 }

60 #endif

stan::math::cumulative\_sum

std::vector< T > cumulative\_sum(const std::vector< T > &x)

Return the cumulative sum of the specified vector.

**Definition:** cumulative\_sum.hpp:23

Eigen.hpp


---

[ Stan Home Page ]

*© 2011–2014,
Stan Development Team.*
